# Fancy shoes and painful feet: Hallux valgus and fracture risk in medieval Cambridge, England

**DOI:** 10.1016/j.ijpp.2021.04.012

**Published:** 2021-12

**Authors:** Jenna M. Dittmar, Piers D. Mitchell, Craig Cessford, Sarah A. Inskip, John E. Robb

**Affiliations:** a McDonald Institute for Archaeological Research, University of Cambridge, Cambridge, UK; b Department of Archaeology, University of Aberdeen, Aberdeen, UK; c Department of Archaeology, University of Cambridge, Cambridge, UK; d Cambridge Archaeological Unit, Department of Archaeology, University of Cambridge, Cambridge, UK; e School of Archaeology and Ancient History, University of Leicester, Leicester, UK

**Keywords:** Bunion, Fall, FOOSH injury, Foot problems, Fashion trends, Social status, Impaired mobility

## Abstract

**Objective:**

Hallux valgus, the lateral deviation of the great toe, can result in poor balance, impaired mobility and is an independent risk factor for falls. This research aims to compare the prevalence of hallux valgus in subpopulations of medieval Cambridge, England, and to examine the relationship between hallux valgus and fractures to examine the impact of impaired mobility and poor balance caused by this condition.

**Materials:**

177 adult individuals from four cemeteries located in Cambridge, England.

**Methods:**

Human remains were macroscopically and radiographically assessed.

**Results:**

Hallux valgus was identified in 18 % of individuals and was significantly more common during the 14th–15th centuries than the 11th–13th centuries. The highest prevalence was observed in the friary (43 %), followed by the Hospital (23 %), the rurban parish cemetery (10 %), and the rural parish cemetery (3%). Fractures from falls were significantly more common in those with hallux valgus than those without.

**Conclusion:**

The increased prevalence of hallux valgus identified in individuals from the 14th to 15th centuries coincided with the adoption of new footwear with pointed toes. Those that adopted this fashion trend appear to have been more likely to develop balance and mobility problems that resulted in an increased risk of falls.

**Significance:**

This is the first study to explore the relationship between foot problems and functional ability by studying hallux valgus in archaeological assemblages.

**Limitations:**

Falls are complex and determining the mechanism of injury in human skeletal remains is not always possible.

**Further research:**

Fracture prevalence rates may have been affected by biological factors and underlying pathological conditions.

## 1 Introduction

Clothing and personal adornment played a role in the recognition and maintenance of social hierarchy during the High and Late medieval periods in England (Smith, 2009). The 14th century brought about an abundance of new styles of dress and footwear in a wide range of fabrics and colors (Piponnier and Mane, 1997). Research by Mays (2005) has shown that osteological analysis can be used to begin to explore social differences that were reflected in fashion trends, specifically in footwear. This study, conducted on a skeletal assemblage in Ipswich, England identified changes in the prevalence rate of hallux valgus, an excessive lateral angulation of the great toe relative to the first metatarsal, that corresponded to the wearing of pointed shoes (Mays, 2005). Although a range of factors can predispose an individual to hallux valgus including genetics, congenital structural variations in the alignment of the metatarsals, and muscle imbalance, the most common cause is wearing tight, ill-fitting footwear with a pointed toe-box (Inman, 1974; Munteanu et al., 2017; Nix et al., 2012). Constrictive shoes cause this condition by exerting a laterally directed force against the medial aspect of the hallux (Menz, 2008; Menz et al., 2016).

The hallux plays an important role in maintaining upper body stability (Hughes et al., 1990) and the lateral deviation of the hallux can impair the transfer of bodyweight which leads to impaired balance and an unstable gait pattern (Glasoe et al., 2010; Mickle et al., 2011). Several modern studies have found poorer lateral stability, poorer coordinated stability, and increased postural sway in individuals with hallux valgus when compared to those without (Menz et al., 2005; Spink et al., 2011). The severity of the lateral deformity affects balance performance. Due to the detrimental impact that hallux valgus has on an individuals’ gait, the condition is an independent causative risk factor for falls (Dolinis and Harrison, 1997; Mickle et al., 2009).

Although hallux valgus has been identified in numerous archaeological assemblages, the impact that the physical consequences of hallux valgus had on the lived experience of members of past societies has not yet been explored. The aim of this paper is to compare the prevalence rates of hallux valgus in subpopulations of medieval Cambridge, to explore the social differences between those subgroups, and to examine the relationship between hallux valgus and fractures to identify the impact of impaired mobility and poor balance caused by this condition. Here we investigate the inhabitants of medieval Cambridge who were buried in a parish cemetery (which was the normative burial site for the majority of the population), a wealthy friary, a charitable institution for the poor, and a rural parish located approximately 6 km from the town.

### 1.1 Medieval Cambridge

By the 13th century, Cambridge was a medium-sized provincial market town with an estimated population of around 3500 in 1279/80 (Casson et al., 2020a, 318). Including outlying settlements at this time, there were 712 properties at least partly used as dwellings (594 messuages, which are plots of land with residential buildings on them, 27 houses and 91 shops) plus 17 other properties (6 stalls or selds and 11 granges or granaries) plus 77 pieces of land (7 curtilages, 9 crofts, 15 areas of urban land and 46 pieces of rural land (Casson et al., 2020a, b). Economic life was based upon farming and river trade up and down the Cam. The River Cam and the Great Ouse gave direct access to the North Sea, which allowed import and export of goods.

The university was founded c. 1208–1210, but it did not become a major force in the town until the late 13th to early 14th century. Cambridge also held a large non-university clerical population, who were based in the Benedictine nunnery of St. Radegund, the Hospital of St John the Evangelist, the outlying Barnwell priory and nearby leprosarium, and a number of friaries that were founded in the 13th century (Andrews, 2006; Rubin, 1987). The community benefited from these religious establishments and the university as the influx of students and clerics affiliated with the colleges likely contributed to a steady demand for food, drink, and services within the town (Lee, 2005). Over time the growing collegiate and religious institutions became increasingly important markets for many products, some of which were quite specialized.

## 2 Materials & methods

### 2.1 Materials

**Fig. 1:**
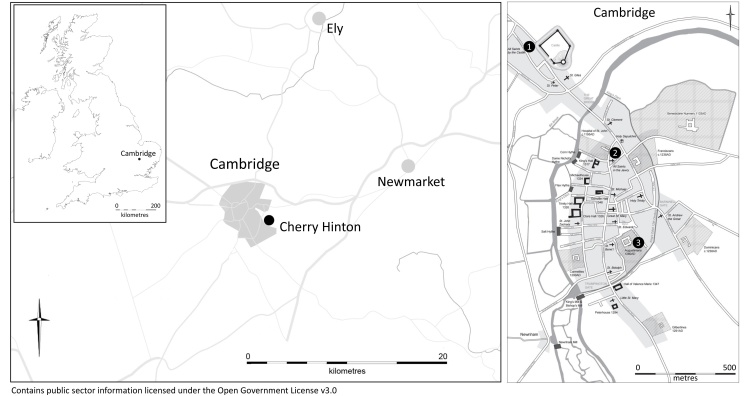
Map of Cambridge and the surrounding area, c. 1350 indicating the location of 1) All Saints by the Castle parish burial ground, 2) the Hospital of St John the Evangelist and 3) the Augustinian friary (Cambridge map by Vicki Herring; UK map inset adapted from image: Uk_outline_map.png).

The human skeletal remains of 177 adult individuals that were buried in four cemeteries located in and around Cambridge (Fig. 1) were analyzed: the parish cemetery of All Saints by the Castle (n = 50), the Hospital of St. John the Evangelist (n = 69), the Augustinian friary (n = 21) and the burial ground associated with the proprietary church at Church End in Cherry Hinton (n = 37). Only individuals over 18 years of age at the time of death with at least one first metatarsal present were included in this study. The individuals from the three sites located within the town represent different social contexts: the rurban (mixed rural and urban) parish cemetery of All Saints by the Castle, represents ordinary, mixed but generally relatively poor townsfolk; the Hospital of St. John the Evangelist predominantly represents inmates of a charitable institution; and the Augustinian friary representing members of the clergy and the relatively wealthy laity. These sites were compared to the individuals buried in the manorial proprietorial church at Church End in Cherry Hinton, a village located 6 km southeast of Cambridge.

#### 2.1.1 The parish church of All Saints by the Castle

The parish of All Saints by the Castle, located north of the river, was likely founded *c*. 940–1100. The parish was used until 1365/6, when it was amalgamated with a neighboring parish following population loss due to the second plague pandemic (which affected Cambridge in 1349). The graveyard of All Saints by the Castle was identified during an archaeological evaluation in 1972 and four areas were excavated in 1973 (Craddock and Gregory, *c*. 1975), with further skeletons uncovered in 1988 and 1994 (Cessford and Dickens, 2005a; Cessford, in press). In total *c.* 215 skeletons in various states of completeness have been excavated. It appears that this parish population comprised a cross-section of social classes, which was probably broadly representative of Cambridge as a whole.

#### 2.1.2 The Hospital of St. John the Evangelist

The Hospital was established *c.* 1190–1200 to provide care for the poor and infirm of Cambridge. It remained in use until it was dissolved to create St. John’s College in 1511 (Underwood, 2008). The detached cemetery was established by *c.* 1204–14 and was excavated during 2010–11 by the Cambridge Archaeological Unit. This excavation unearthed 400 complete and partial in situ burials (Cessford, 2015). The cemetery population consisted of both male and female burials but with no individuals under the age of five (Cessford, 2015:101). Those buried there were probably predominantly hospital inmates, who were poor and unable to be cared for by family members. The cemetery also likely contained a number of ‘corrodians’, who were wealthy elderly lay people often without close relatives who paid to live in a religious institution for the rest of their lives, as well as few local benefactors who made small to moderate donations to the Hospital (Cessford, in press).

#### 2.1.3 The Augustinian friary

This friary was established around 1279/80–89 and continued in use until the Dissolution of the Monasteries in 1538. It functioned as a *studium generale*, or national and international study house, for the Augustinian order in England (Roth, 1966). The friary acquired the right to bury members of the Augustinian order in 1290 and individuals who were not members of the friary in 1302 (Cessford, in press). Parts of the friary were excavated in 1908-09, during which human skeletal remains from *c.* 47 individuals were recovered. As only the skulls remain available for analysis, these individuals were not included in this study. In 2016–17 the Cambridge Archaeological Unit excavated other parts of the site, and 38 further single inhumations burials were uncovered (Cessford, 2017). From this excavation, 32 skeletons were excavated from the cemetery to the south of the friary church dating to *c.* 1290–mid/late 14th century, and six in a chapter house of *c*. mid/late 14th century–1538 (Cessford, 2017). These individuals represent a mixture of members of the Augustinian order and lay benefactors. These burials were differentiated archaeologically as friars appear to have been buried clothed and wearing a belt with buckle; whilst lay people were typically buried wrapped in a shroud. The townspeople buried in the friary likely represent some of the more prosperous members of Cambridge society as typically burial within the friary was secured through donations (Cessford, in press). Both friars (n = 11) and laypeople (n = 10) were included in this study.

#### 2.1.4 The proprietary church at Church End, Cherry Hinton

Located around six kilometers southeast of Cambridge, the church and cemetery at Church End, Cherry Hinton, was excavated by the Hertfordshire Archaeological Trust in 1999 (Cessford and Dickens, 2005b; Lally, 2008; McDonald and Doel, 2000). Over 670 graves were excavated, with the remains of c. 980 in situ and disarticulated individuals present. Of these, a sample of individuals that comprised 37 adults with surviving feet were analysed. The burials at Cherry Hinton were west-east aligned extended supine inhumations, in simple earth cut graves with the head to the west and most individuals were probably buried in shrouds. Apart from four adults with accompanying neonates, there were no identifiable multiple burials. There were a range of rites, including anthropomorphic graves with distinctive head 'niches', head support stones, evidence of a few possible coffins and a few clothed burials. Disarticulated human remains were present in some grave fills, but these were not included in the study. The cemetery was associated with a rural estate center starting around 940–1000 and continuing in use until *c*. 1120–70. Those buried are a broadly representative cross-section of the various types of free and unfree peasants of this predominantly agricultural site.

### 2.2 Methods

The human skeletal remains in this study were assessed following internationally accepted guidelines (Buikstra and Ubelaker, 1994; Mitchell and Brickley, 2017) as part of the ‘After the Plague: Health and History in Medieval Cambridge’ project. Each skeleton was assigned a “project specific number” (PSN) that served as a unique identifier. Individuals are referred to by these numbers throughout. The biological sex of each adult skeleton was estimated by examining the sexually dimorphic characteristics of the pelvis and cranium (Buikstra and Ubelaker, 1994; Phenice, 1969; Schwartz, 2007) and, when available, through aDNA analyses (see Inskip et al., 2019). Age-at-death was estimated using the degenerative changes observable on the pubic symphysis (Brooks and Suchey, 1990); auricular surface (Buckberry and Chamberlain, 2002), sternal rib ends (İşcan et al., 1984, 1985) and the sternal end of the clavicle (Falys and Prangle, 2015). Skeletons were divided into the following age categories: young adult (18–25 years), middle adult (26–44 years), mature adult (45–59 years), and old adult (60+ years). If age-at-death could not be determined due to incompleteness or damage, individuals with complete epiphyseal fusion were classified as ‘adult’. Table 1 displays the sex and age-at-death distribution for all of the individuals included in this study.

**Table 1:** Frequency table for biological sex and age-at-death categories by site.

Age	Male	Female	Unobservable, sex unknown	Total
**All Saints by the Castle**				
Young adult	2	1	0	3
Middle adult	6	4	0	10
Mature adult	7	5	0	12
Old adult	5	5	1	11
Adult	1	0	13	14
**Total**	**21**	**15**	**14**	**50**

**Hospital of St. John the Evangelist**				
Young adult	3	5	0	8
Middle adult	14	5	0	19
Mature adult	10	5	0	15
Old adult	3	1	0	4
Adult	1	2	20	23
**Total**	**31**	**18**	**20**	**69**

**Augustinian friary**				
Young adult	2	0	0	2
Middle adult	7	0	0	7
Mature adult	4	0	0	4
Old adult	2	1	0	3
Adult	1	0	4	5
**Total**	**16**	**1**	**4**	**21**

**Church End, Cherry Hinton**				
Young adult	0	0	0	0
Middle adult	8	7	0	15
Mature adult	8	3	0	11
Old adult	1	1	0	2
Adult	3	1	5	9
**Total**	**20**	**12**	**5**	**37**

As part of the paleopathological assessment, the pedal skeletal elements of the great toe were macroscopically assessed for evidence of hallux valgus based on the criteria outlined in Mays (2005). Long standing lateral subluxation of the great toe is characterized by:

1) The presence of a ridge of bone within the footprint of the articular surface of the metatarsal head, a fifth or a quarter of the distance across the normal joint surface. This ridge corresponds with the margin of articulation of the mal-aligned proximal phalanx of the toe
2) Subluxation of the metatarsal head relative to the sesamoid bones can lead to erosion and lipping at the inter-sesamoid ridge on the underside of the metatarsal head.
3) Inflammation at the attachment of the collateral ligaments at the metatarsophalangeal joint and in the adjacent bursa can lead to superficial erosion and cavitation of the medial aspect of the metatarsal head
4) Degenerative changes to the base of the tarsometatarsal joint of the first metatarsal
5) Degenerative changes on the head of the first metatarsal

To examine the relationship between hallux valgus and injury due to falls, the presence of fractures was compared between the groups of individuals with hallux valgus and those without. As the objective of this assessment is to establish the relationship between accidental injury due to falls from a standing position, weapon injuries and skeletal trauma to the splanchnocranium (including dental trauma) were not included in this analysis. Cases of spondylolysis and os acromiale were not counted as skeletal trauma. In order to be included in this assessment, a skeleton was required to be over 25 % complete and well preserved with minimal evidence of taphonomic damage to the bone cortex. When appropriate, Fisher's exact tests were used to determine statistical significance. Significance level was set at p < .05.

Falls are one of the most common mechanisms for fractures across all age groups. In the clinical research, up to 80 % of fractures are attributed to falls, especially in older patients (Bergdahl et al., 2016). Falls on outstretched hand (FOOSH) injuries are some of the most commonly identified fractures associated with falls. These include fractures located on the distal radius, radial or ulnar styloid process, distal radioulnar joint, head of the radius, the scaphoid, the hook of the hamate, the clavicle and the humeral head (Bartuseck, 2018). Within this research, a fracture to the humerus, radius, ulna, clavicle, the acromion and/or scapular spine of the scapula or any of the carpal bones were considered to be potentially caused by the FOOSH mechanism. However, hip and ankle fractures are also common in same level falls, and fractures to the vertebral column, ribs and skull have also been reported (Aharonoff et al., 2003; Barrett-Connor et al., 2010; Nevitt and Cummings, 1992; Old and Calvert, 2004). As fractures that occur from falls are highly variable, fractures located anywhere on the post-cranial skeleton were also explored statistically to identify any difference between subgroups.

A fracture was classed as antemortem if healing was present. If no evidence of healing was present, the fracture was classed as perimortem. Perimortem fractures were distinguished from postmortem damage based on the coloration of the bone at the fracture, the location of the injury and the fracture morphology (Galloway and Wedel, 2014; Sauer, 1998). Selected fractured elements were analysed using plain radiography (x-ray). X-rays were taken by Reveal X-ray Imaging Solutions using a portable DR-Go x-ray machine.

Each fracture was described and classified according to Lovell (1997). When analysing skeletal trauma, the causative mechanisms (direct, indirect, stress, secondary to pathology) and the forces applied to a bone can be inferred by the type of break that has occurred: transverse, oblique, spiral, comminuted, impacted, greenstick, avulsion or compression (see Galloway and Wedel, 2014; Redfern and Roberts, 2019). Transverse, comminuted and compression fractures often occur as the result of direct trauma (Lovell, 1997). Transverse fractures run perpendicular to the shaft and are caused by a combination of tension and compression stresses; they are often associated with high energy mechanisms or direct force but can also occur as the result of a fall (Rogers and Waldron, 2001). Comminuted fractures, where the bone fractures into multiple fragments, commonly result from high impact mechanisms. Compression fractures occur as the result of a sudden impaction (Redfern and Roberts, 2019). Oblique, spiral, impacted, greenstick, and avulsion fractures result from indirect trauma (Lovell, 1997). Oblique fractures extend diagonally across the bone (with an angle ≥30° perpendicular to the long axis) and are caused by compressive and shearing stresses (Galloway and Wedel, 2014; Meinberg et al., 2018). Spiral fractures are complete fractures caused by torsion (an indirect mechanism), which causes the bone to twist apart (Galloway and Wedel, 2014). Oblique and spiral fractures are indicative of higher energy mechanisms and are often related to falls or jumps from a height (Johner et al., 2000). An impacted fracture is a complete fracture where the broken ends are driven into each other so that the fracture line is indistinct. Such fractures usually result from compression (Galloway and Wedel, 2014). Greenstick fractures are incomplete fractures in immature individuals that affect only one side of a bone as the result of bending from the opposite side. Avulsion fractures occur when a tendon or ligament pulls off a piece of bone.

However, classification of fractures according to these types is not always straightforward as fractures may be the result of more than one type of stress. Within archaeological assemblages, classification is further complicated by remodeling at the site of a fracture, which may prevent the identification of a specific type. In cases where substantial healing occurred and the fracture line was no longer visible, the fracture type was classed as unobservable. The type and pattern of fractures present on each individual were considered and the possible mechanisms for each injury was inferred.

It is well known that age-related loss of bone mass increases the likelihood of fractures in older individuals (Cummings and Melton, 2002). However, there are many limitations of diagnosing osteopenia and osteoporosis in archaeological human skeletal remains. As such, no attempt was made to identify individuals within these assemblages that may have had these conditions.

#### 2.2.1 Dating methods

The skeletons have all been dated on the basis of a combination of the overall periods when the burial grounds are documented as being in use, plus the skeletons’ stratigraphic location with the burial sequences at the sites. Those from the Hospital of St John the Evangelist have also been radiocarbon dated. In addition to standard calibration, allowance has been made for various factors that can affect radiocarbon determinations on human bone making them appear older than the date an individual died and that are not covered by standard calibration, such as marine dietary offset and bone turnover during life (for a full methodological description see Cessford and Alexander, in press).

## 3 Results

**Fig. 2:**
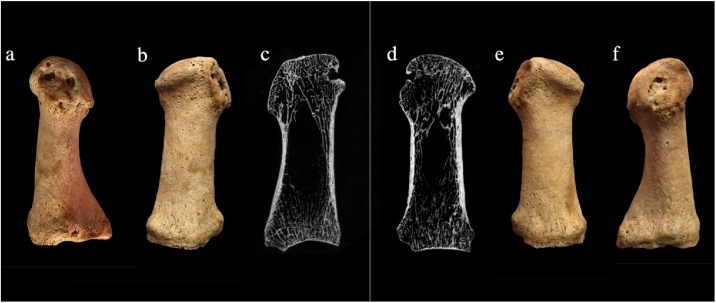
Lesions associated with hallux valgus (PSN 525) in an adult female individual from the Augustinian friary. a) and f) medial aspect of the first metatarsals showing lytic cavitation at attachment of collateral and sesamoid ligaments, b) and e) dorsal view of first metatarsals showing the lateral deviation of the articular surface, c) and d) μCT images showing lytic lesions on medial side of the metatarsal heads. Photographs taken by Jenna Dittmar, μCT scans taken by Bram Mulder.

Changes at the metatarsophalangeal joint consistent with longstanding hallux valgus (Fig. 2) were identified in 18 % (n = 31/177) individuals (see Table 2). The majority of individuals with hallux valgus were male (n = 20). The highest prevalence rate of 45 % (n = 5/11) was identified in the friars buried in the Augustinian friary, followed by the layfolk buried in the friary (40 %, n = 4/10); 23 % (n = 16/69) of the individuals buried in the burial ground of the Hospital of St. John had hallux valgus, as did 10 % (n = 5/50) of those buried in the All Saints by the Castle parish burial ground. The prevalence of hallux valgus in the individuals buried within the rural cemetery at Cherry Hinton was 3% (n = 1/37). When examined statistically, significantly more individuals from the town had hallux valgus than did those from the rural hinterland (p = .01).

**Fig. 3:**
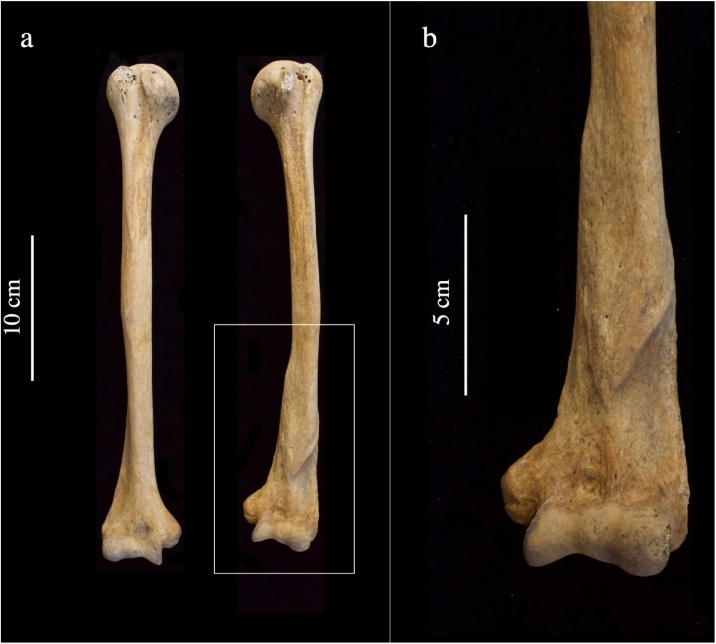
a) Anterior view of humerii of from a mature adult female (PSN 357) with co-occurring hallux valgus and hallux rigidus buried in the burial ground of the Hospital of St John the Evangelist in Cambridge, b) close-up of antemortem fracture of distal left humerus. Photographs by Jenna Dittmar.

**Table 2:** Age and biological sex distribution of individuals with hallux valgus by site.

Age	Male	Female	Unobservable, sex unknown	Total	Prevalence by age cat.
**All Saints by the Castle**					
Young adult	0/2	0/1	0/0	0/3	0%
Middle adult	1/6	0/4	0/0	1/10	10 %
Mature adult	1/7	2/5	0/0	3/12	25 %
Old adult	1/5	0/5	0/1	1/11	9%
Adult	0/1	0/0	0/13	0/14	0%
**Total**	3/21	2/15	0/14	5/50	10 %
**Prevalence by sex cat.**	14 %	13 %	**0%**	10 %	

**Hospital of St. John the Evangelist**					
Young adult	0/3	0/5	0/0	0/8	0%
Middle adult	3/14	0/5	0/0	3/19	16%
Mature adult	5/10	2/5	0/0	7/15	47%
Old adult	1/3	0/1	0/0	1/4	25 %
Adult	1/1	0/2	4/20	5/23	22%
**Total**	10/31	2/18	4/20	16/69	23 %
**Prevalence by sex cat.**	32 %	11 %	20 %	23 %	

**Augustinian friary**					
Young adult	2/2	0/0	0/0	2/2	100 %
Middle adult	2/7	0/0	0/0	2/7	29%
Mature adult	2/4	0/0	0/0	2/4	50%
Old adult	1/2	1/1	0/0	2/3	67%
Adult	0/1	0/0	1/4	1/5	20 %
**Total**	7/16	1/1	1/4	9/21	43 %
**Prevalence by sex cat.**	44 %	100 %	25 %	43 %	

**Church End, Cherry Hinton**					
Young adult	0/0	0/0	0/0	0/0	0%
Middle adult	0/8	1/7	0/0	1/15	7%
Mature adult	0/8	0/3	0/0	0/11	0%
Old adult	0/1	0/1	0/0	0/2	0%
Adult	0/3	0/1	0/5	0/9	0%
**Total**	0/20	1/12	0/5	1/37	**3%**
**Prevalence by sex cat.**	**0%**	**8%**	**0%**	**3%**	

Using a combination of the overall periods when the burial grounds are documented as being in use, the skeleton’s stratigraphic location with the burial sequences at the sites, radiocarbon dating and Bayesian modelling we were able to divide 123 individuals into distinct temporal categories: those that date to 11th–13th centuries (n = 52) and those to the 14th -15th centuries (n = 71). Hallux valgus was significantly more prevalent during the 14th -15th centuries (n = 19/71) than in the 11th–13th centuries (n = 3/52) (p = .003).

### 3.1 Hallux valgus & fracture prevalence

A total of 159 individuals were complete enough to be assessed for skeletal trauma (male = 86, female = 47, unknown sex = 26). Of those, 34 % (n = 55/159) individuals had one or more fractures. Nearly all of the fractures observed in this assemblage occurred antemortem; only one individual (PSN 531) had peri-mortem fractures (see Table 6; or Dittmar et al., 2021 for further details). More males (44 %, n = 38/86) had fractures than did females (32 %, n = 15/47). The location and patterning of the skeletal trauma present within this sample is largely consistent with injuries that reflect hazards associated with the individual’s living environment, such as falls as well as occupational-related trauma. However, several individuals within these assemblages had fractures in locations that are commonly associated with interpersonal violence (i.e. cranial vault, mandible, ribs) (see Dittmar et al., 2021).

Of the individuals with hallux valgus, 52 % had one or more fractures (n = 15/29), whilst 31 % of those without hallux valgus had evidence of a fracture (n = 40/130). When assessed using a Fisher’s exact test, it was found that a significantly greater proportion of individuals with hallux valgus had fractures than did those without hallux valgus (p = .03). A higher percentage of females with hallux valgus had fractures (80 %, n = 5/6) than did males with hallux valgus (45 %, n = 9/20) (Table 3). When examined by age category, the frequency of fractures increased with age in both males and females (Table 3).

**Table 3:** Age and sex distribution of individuals with fractures for those with and without hallux valgus.

	# of individuals with fractures with HV	# of individual with fractures without HV	Total
**Male**			
Young adult	0/2	1/4	1/6
Middle adult	2/6	9/28	11/34
Mature adult	5/9	10/20	14/29
Old adult	2/2	8/11	10/13
Adult	0/1	1/3	1/4
**Total**	**9/20**	**29/66**	**38/86**

**Female**			
Young adult	–	1/6	1/6
Middle adult	0/1	3/15	3/16
Mature adult	3/3	4/11	7/14
Old adult	2/2	2/6	4/8
Adult	–	0/3	0/3
	**5/6**	**10/41**	**15/47**

**Unknown**			
Young adult	–	–	–
Middle adult	–	–	–
Mature adult	–	–	–
Old adult	–	0/1	0/1
Adult	1/3	1/22	2/25
**Total**	**1/3**	**1/23**	**2/26**
**TOTAL**	**15/29**	**40/130**	**55/159**

Fractures to the axial skeleton (n = 42/159) was more common than trauma to the appendicular skeleton (n = 34/159) (Table 4). Multiple trauma that affected the axial and appendicular skeleton was observed on 13 % (n = 21/159) of individuals. Fractures to the ribs and to the bodies of the vertebra were the most commonly recorded fractures to the axial skeleton (Table 5).

**Table 4:** Location of trauma on individuals with hallux valgus and those without.

	Individuals with HV			Individuals without HV		
Sex	Appendicular	Axial	Both	Appendicular	Axial	Both
Male	1	5	3	8	9	12
Female	2	–	3	1	6	3
Unknown	1	–	–	–	1	–
**TOTAL**	**4**	**5**	**6**	**9**	**16**	**15**

**Table 5:** Location of trauma to the postcranial axial skeleton present on individuals with hallux valgus and those without.

	Individuals with HV		Individuals without HV	
Sex	Rib(s)	Vertebral body (compression)	Rib(s)	Vertebral body (compression)
Male	4/19	2/17	14/58	4/47
Female	3/3	1/6	6/38	6/33
Unknown	–	–	1/4	1/2
**Total**	**7/22**	**3/23**	**21/100**	**11/82**

#### 3.1.1 FOOSH injuries & Fractures from falls

Fractures to the upper limb that could have resulted from a fall forward onto an outstretched arm were observed in 15 individuals (Table 6). One individual (PSN 353) had a supracondylar humerus fracture, a type of fracture that typically occurs during childhood, most commonly in the first decade of life. Supracondylar fractures occur through the distal humerus, at the level of the olecranon fossa, and are the result of a fall onto an outstretched hand in 97%–99% of cases (Vaquero-Picado et al., 2018). However, as the aim of this research is to examine the relationship between hallux valgus and fractures, the injuries present on PSN 353 were not included as evidence of a fall in this analysis because of the age at which this fracture occurred. A number of other fractures that were likely the result of a fall were also identified within this sample (Table 6). An adult male from the Augustinian friary had a well healed coccyx fracture that likely occurred in a backwards fall and two individuals had healed hairline fractures on the articular surface of their patellae that likely resulted from a fall onto the knees. In addition to this, antemortem fractures to the femoral necks (intracapsular fractures) were identified on two old adult females (PSN 335 and PSN 729). This type of fracture can occur as a result of low energy falls from standing height with a backwards or side impact to the hip, especially in older adults (Hayes et al., 1993).

**Table 6:** Interpretation (based on location and type) of fracture(s) present in individuals that had one or more fractures that may have occurred as the result of a fall. All fractures in this table occurred antemortem. Fracture type was recorded as unobservable in cases of extensive healing.

PSN	Site	Sex	Age	Element	Side	Location of fracture	Fracture Type	Rib(s) Fractures	Compression fractures	Mechanism and interpretation
**Individuals with Hallux Valgus**										
49	Hospital of St. John	M	Mature adult	Femur	Right	Distal condyle	Hairline	–	–	Direct trauma, fall onto knees
				Patella	Left	Articular surface, inferior aspect	Hairline			
335	Hospital of St. John	F	Mature adult	Femur	Right	Neck	Unobservable	X	X	Indirect trauma, likely secondary to age-related bone loss
				Patella	Right	Articular surface	Hairline			Direct trauma, possible fall onto knee
				Ulna	Right	Styloid process	Transverse, non-united			Indirect trauma, fall onto outstretched hand
				Radius	Right	Distal ¼ of shaft	Oblique			Indirect trauma, fall onto outstretched hand
357	Hospital of St. John	F	Mature adult	Humerus	Left	Distal 1/3 of shaft	Spiral	X	–	Indirect trauma, fall on to outstretched hand (see Fig. 3)
510	Augustinian friary (Chapter house)	M	Adult	Ulna	Left	Distal 1/3 of shaft	Transverse	–	–	Possibly direct trauma, from a direct blow to forearm, could also be associated with a fall
				5^th^ metacarpal		Base	Oblique			Likely indirect trauma, from a fall on a flexed wrist with the arm in extension and axial loading of the metacarpal. Could also be direct trauma, but less likely.
729	All Saints parish	F	Old adult	Femur	Right	Neck	Unobservable, non-united fracture with extensive remodeling	–	–	Possibly direct trauma as the result of a fall, likely secondary to osteoporosis
738	All Saints parish	M	Old adult	Fibula	Right	Proximal 1/3 of shaft	Oblique	–	X	Likely direct trauma, may be related to a fall or the result of a lateral blow
				Clavicle	Right	Midshaft	--			Indirect trauma, fall onto outstretched hand but could also be direct trauma to shoulder
				Scapula	Right	Acromion process	--			Likely indirect trauma, fall onto outstretched arm but could also be direct trauma to shoulder
772	All Saints parish	M	Young adult	Ulna	Left	Distal 1/3 of shaft	Unobservable, likely transverse	X	–	Likely indirect trauma, fall onto outstretched arm but could also be direct trauma
				5^th^ Metacarpal	Right	Base (proximal metaphysis and proximal articular surface)	Oblique			Likely indirect trauma from a fall on a flexed wrist with the arm in extension and axial loading of the metacarpal. Could also be direct trauma, but less likely.

**Individuals without Hallux Valgus**										
84	Hospital of St. John	M	Young adult	Ulna	Right	Distal ¼ of shaft	Spiral	–	–	Indirect trauma, fall onto an outstretched hand or direct trauma
103	Hospital of St. John	M	Middle adult	Scapula	Left	Acromion process	--	X	–	Likely direct trauma to the shoulder (left 2^nd^ rib also fractured), but could be from indirect trauma, fall onto an outstretched hand
156	Hospital of St. John	M	Young adult	Ulna	Left	Midshaft	Unobservable	–	–	Direct or indirect trauma, fall onto an outstretched hand
				5^th^ metatarsal	Right	Tuberosity of 5^th^ metatarsal	Avulsion			Indirect trauma, caused by the forcible inversion of the foot in plantar flexion
353	Hospital of St John	M	Old adult	Humerus	Left	Supracondylar fracture	Unobservable	–	–	Fall on to outstretched hand during childhood
520	Augustinian friary	M	Middle adult	Coccyx	--	---	--	–	–	Direct trauma, fall onto backside
531 a	Augustinian friary	M	Middle adult	Clavicle	Left	Distal	--	–	–	Direct trauma, most commonly from a fall onto the shoulder
718	All Saints parish	F	Mature adult	Ulna	Left	Distal 1/4 of shaft	Unobs.	X	X	Indirect trauma, fall onto an outstretched hand
730	All Saints parish	M	Middle adult	Radius	Right	Distal 1/4 of shaft	Transverse	–	Unobs	Indirect trauma, fall onto an outstretched hand
				Ulna	Right	Styloid process	Transverse			Indirect trauma, fall onto an outstretched hand
919	Proprietary Church, Cherry Hinton	M	Middle adult	Radius	Right	Distal ¼ of shaft	Oblique	X		Indirect trauma, fall onto an outstretched hand
923	Proprietary Church, Cherry Hinton	M	Mature adult	Radius	Right	Distal ¼ of shaft	Oblique	X	Unobs	Indirect trauma, fall onto an outstretched hand
				Fibula	Right	Proximal ½ of shaft	Oblique			Likely direct trauma, may be related to a fall or the result of a lateral blow
943	Proprietary Church, Cherry Hinton	M	Mature adult	Ulna	Left	Midshaft	Oblique	X		Indirect trauma, fall onto an outstretched hand

a PSN 531 also had perimortem bilateral comminuted femoral fractures in addition to perimortem fractures of T1 and C6 that indicate that he was involved in a severe, likely lethal, accident (see Dittmar et al., 2021). These fractures were not included in this table as this event is unlikely to be related to a fall.

A total of 11 % (n = 17/159) of individuals in this sample sustained one or more fractures that were likely the result of a fall. Of these, seven had hallux valgus, ten did not. When assessed using a Fisher’s exact test, it was found that individuals with hallux valgus were significantly more likely to have a fracture that likely resulted from a fall than were individuals without hallux valgus (p = .02). When examined by age group, individuals in the young (18–25 years) and middle adult (18–44 years) age categories were combined due to low numbers in certain age categories, as were the mature (44–59 years) and old adult (60+ years) age categories. This analysis revealed that there was no difference in the frequency of fractures that occurred from falls between young/middle adults with hallux valgus and those without hallux valgus (p = 1), but this relationship was significant in the mature/old adult age group (p = .03). The sample size precluded the exploration of these relationships by biological sex.

## 4 Discussion

Hallux valgus was present in at least 18 % of the individuals examined within this study. Due to the progressive nature of the condition, it is likely that this an underestimate, as typically only longstanding moderate to severe cases are associated with skeletal changes. We did note a further 6% of individuals had areas of lytic change on the medial side of the metatarsal heads, but no changes on the articular surface itself. These could indicate early cases of hallux valgus that had not yet developed joint surface changes, or alternatively they might fall within the normal range of variation for the human foot. Additional research into the osteological changes associated with less severe cases of hallux valgus is required in order to explore these questions further.

Although there are a range of factors that can predispose an individual to hallux valgus, including age, gender, obesity, and congenital structural variation, one of the most commonly cited causative factors is tight ill-fitting footwear with a pointed toe-box (Menz et al., 2016; Munteanu et al., 2017). The substantial differences seen in the prevalence rate of hallux valgus between the burial locations within Cambridge and across time periods indicate that the differences observed are not due solely to congenital variation or genetic factors. We would argue that these differences are likely reflective of social factors that exist within and between subgroups of Cambridge society.

**Fig. 4:**
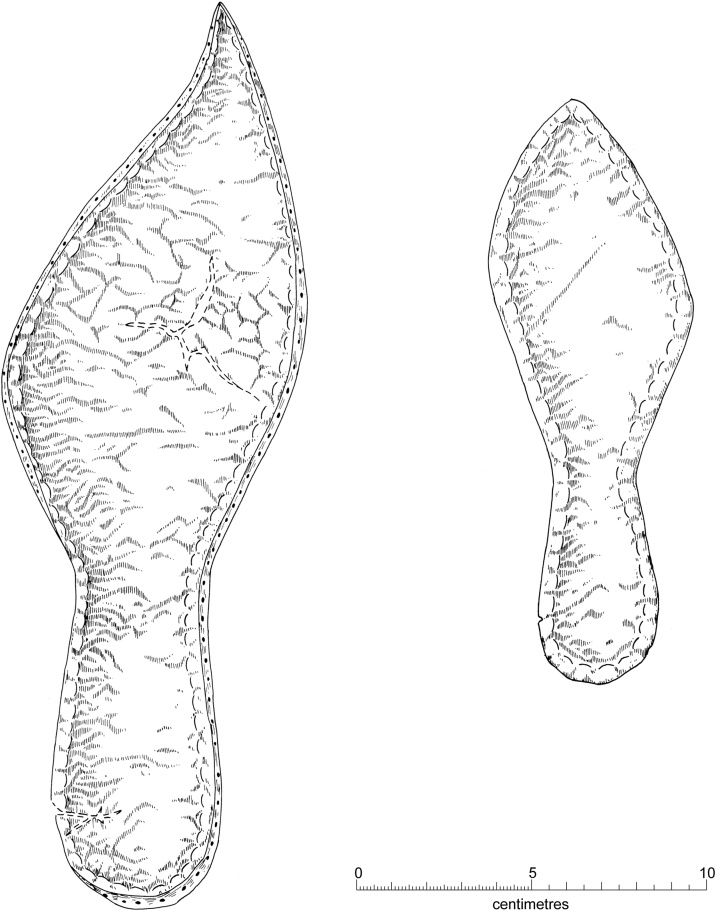
Fourteenth century leather shoes from the King’s Ditch, Cambridge. Left: turnshoe sole, adult left foot; Right: turnshoe sole, child’s right foot. Previously published in Cessford and Dickens (2019), Fig. 3.12D and 12F. Copyright Cambridge Archaeological Unit.

The 14th century brought about an abundance of new styles of dress and footwear in a wide range of fabrics and colors (Piponnier and Mane, 1997). Amongst the new fashion trends were pointed shoes with lengthy tips or “poulaines” (Fizzard, 2007). The remains of shoes excavated from the Baynard’s Castle site in London showed that while a rounded toe box was relatively common in the early 14th century, by the late 14th century almost every type of shoe was at least slightly pointed (Cessford and Dickens, 2019; Grew and de Neergaard, 1988). Although more limited in number, shoes from Cambridge (Fig. 4) appear to follow a similar pattern with clear evidence for the adoption of pointed styles by both adults and children alike (Cessford and Dickens, 2019).

This transition in footwear is consistent with the significantly higher prevalence rate of hallux valgus in Cambridge from the 14th to 15th centuries compared to those that lived during the 11th–13th centuries. These findings are similar to previous research on hallux valgus undertaken on a skeletal assemblage from Ipswich, UK. That study found no osteological evidence for the hallux valgus in those that lived from the ninth to eleventh centuries (n = 47 adults), but it was observed in 7% of individuals from 13th to 16th centuries (n = 14/192) (Mays, 2005).

This fashion trend appears to have not been uniformly adopted by all members of Cambridge society, as substantial variation in prevalence exists between the individuals buried in different locations within the town boundaries. The prevalence of hallux valgus in the hospital inmates (20 %), suggests that the individuals buried there were more likely to don such apparel than were the individuals buried in the rurban (areas with a mixture of urban and rural activities) cemetery of All Saints by the Castle (10 %). This difference in prevalence rate is likely partly explained by the use dates of the cemetery of All Saints by the Castle, which went out of use in 1365. Unfortunately, it was not possible to examine temporal differences at this site.

The highest prevalence rate was observed in the Augustinian friary (43 %), which suggests that fashion trends in footwear were embraced by wealthy laypeople and members of the clergy alike. As a mendicant order that adopted a lifestyle of poverty, the Augustinian friars had strict rules regarding attire that was considered appropriate. Footwear, for example was supposed to be ‘black and fastened by a thong at the ankle’ (Gutiérrez, 1984, vol. I: 63). In the 13th and 14th centuries it was becoming progressively more common for those in the clerical orders in Britain to wear stylish clothes and footwear, a point which was the cause of much concern and dissuasion amongst the higher-ranking church officials. Despite the 4th Lateran Council of 1215 forbidding the clergy from wearing pointed toe or embroidered shoes, we nevertheless hear of Augustinian Canons (a different order to Augustinian friars) who wore ‘pointed toed shoes laced in various ways’, and the order was criticized for its members wearing ‘fashionable tight shoes’ rather than boots (Fizzard, 2007). Following this, the church passed numerous decrees in response to continued indiscretions regarding clerical dress, most notably in 1281 and 1342 (Wilkins, 1737, vol. 2: 4, 59–60, 703). The adoption of fashionable garments by members of the clergy was so common that it spurred criticism in contemporary literature, as is seen in Chaucer’s depiction of the monk in *The Canterbury Tales* (Chaucer, 2011).

The mean prevalence of hallux valgus in those buried in these four cemeteries in Cambridge was substantially higher than the prevalence rate in their rural counterparts from Cherry Hinton, with 14 % versus 3% respectively. The difference observed between the Cambridge and rural group in this study is partly explained by the use dates of the cemetery in Cherry Hinton (c. 940/1000 – 1120/70), which is earlier than the majority of the sites within Cambridge. This burial ground went out of use prior to the wide adoption of pointed shoes, and it is likely that those residing in the parish predominately wore soft leather shoes, or possibly went barefoot. Future research that explores hallux valgus in archaeological assemblages would enable further discussion about the adoption of footwear in different subsets of medieval society.

### 4.1 Hallux valgus and risk of fractures

The location and patterning of the skeletal trauma in this sample is largely consistent with injuries that reflect hazards associated with the individual’s living environment, such as falls, as well as occupational and age-related trauma. Individuals with hallux valgus were significantly more likely to have one or more fractures than were those that did not have hallux valgus. When explored further, no relationship between hallux valgus and fractures to the axial skeleton were identified. However, individuals with hallux valgus were significantly more likely to have a fracture that likely resulted from a fall than were individuals without hallux valgus. Overall, the findings of this research suggest that the adoption of fashionable footwear, triggering hallux valgus, was associated with higher prevalence rates of fractures within certain subsets of the Cambridge population, particularly in those who died at an advanced age.

This is consistent with modern clinical research that shows that hallux valgus is associated with impaired mobility, loss of the ability to maintain balance, and is a causative risk factor for falls that is independent of age (see Glasoe et al., 2010; Mickle et al., 2011). Menz et al. (2005) has also shown that the detrimental gait alterations caused by hallux valgus contributes to an increased risk of falling especially in older people when walking on irregular ground. A finding that was mirrored in this research: those with hallux valgus who died over the age of 45 (n = 10/14) were more likely to have antemortem skeletal trauma than younger adults with hallux valgus (n = 2/9). This finding may be partly explained by biological factors such as age-related bone loss which contribute to the higher prevalence rate of antemortem fractures in older adult individuals as weaker bones are more prone to fracture during a fall (Johnell and Kanis, 2005; Riggs et al., 2006).

### 4.2 Limitations of research

The causes of falls are complex and most are multi-factorial in origin. In addition to the traditional risk factors for falls (e.g. age, impaired balance, etc.), the fall descent, fall impact, and bone strength are all important determinants of whether a fracture will occur or not (Berry and Miller, 2008). While 40–60 % of falls lead to injuries, fractures only occur in approximately 5–10 % of falls (Berry and Miller, 2008; Nevitt et al., 1991). As most falls do not result in a fracture and soft tissue injuries are often not possible to assess, the full extent of the consequences of balance issues caused by hallux valgus will not be evident in the archaeological record. These issues are compounded by difficulties in assessing ante-mortem skeletal trauma. Depending on the success of the healing process, it is not always possible to classify the type of fracture, infer the mechanism of injury or to determine the timing of well-healed skeletal trauma.

A key limitation of all studies that analyze and interpret fractures in human skeletal remains is that skeletal trauma accumulates over the course of an individuals’ life. Every traumatic event that did not result in the death of the individual, can potentially leave observable evidence of a fracture. In general, this means that those that lived longer will have had more opportunities to experience antemortem injuries and to accumulate evidence of these injuries. Biological changes experienced by older adult individuals such as age-related loss of bone mass will also increase an individuals’ risk for fractures (Cummings and Melton, 2002). This combination of factors may have contributed to the higher prevalence rate of fractures in older adult individuals observed in this study. It is also possible, and likely that fracture prevalence rates in this study were affected by underlying pathological conditions (see Ives and Brickley, 2014; Judd and Roberts, 1998), early-life health (Parsons et al., 1997), nutrition (Weisz and Albury, 2013), and reproductive behaviors (Mays, 2006).

## 5 Conclusion

Hallux valgus was present in at least 18 % of the individuals examined within this study. Due to the nature of the condition, it is likely that this an underestimate as typically only longstanding cases that are classed as moderate to severe are associated with skeletal changes. The prevalence of hallux valgus was highest in the Augustinian friary, moderate in the other townsfolk, and lowest in the rural parish cemetery. When explored temporally, hallux valgus was significantly more prevalent during the 14th -15th centuries than during the 11th–13th centuries. This change coincides with the adoption of new fashionable footwear with pointed toes that took place during the 14th century.

Far from being a mild inconvenience, this study has shown that individuals with hallux valgus were significantly more likely to have experienced a fracture that occurred as the result of a fall than those who did not have hallux valgus. Since hallux valgus is known to be an independent risk factor for falls in modern patients (Mickle et al., 2009), it is quite likely to have been the same in the medieval period. This is the first study that has explored the relationship between foot problems with balance and functional ability in archaeological human skeletal remains. Overall, the findings of this research would be compatible with the hypothesis that foot problems caused by the adoption of fashionable footwear had an impact on mobility and balance that resulted in an increased risk of falls within the community of medieval Cambridge.
